# Chemoresistance to doxorubicin induces epithelial-mesenchymal transition via upregulation of transforming growth factor β signaling in HCT116 colon cancer cells

**DOI:** 10.3892/mmr.2015.3356

**Published:** 2015-02-16

**Authors:** JINPENG LI, HAO LIU, JIEPING YU, HONGGANG YU

**Affiliations:** Department of Gastroenterology, Renmin Hospital of Wuhan University, Wuhan, Hubei 430060, P.R. China

**Keywords:** colon cancer, doxorubicin chemoresistance, epithelial-mesenchymal transition, RNA interference, transforming growth factor β/Smad4 signaling

## Abstract

Doxorubicin (Dox) is a commonly used chemotherapeutic drug in human colon cancer. However, it becomes increasingly ineffective with tumor progression, the underlying mechanism of which remains to be elucidated. Emerging evidence has led to the identification of an association between chemoresistance and the acquisition of epithelial-mesenchymal transition (EMT) in cancer. However, it remains to be elucidated whether this process is involved in the development of resistance to Dox in colon cancer. In HCT116 human colon cancer cells treated with Dox (50 nmol/l), EMT was induced, and transforming growth factor (TGF)β signaling and multi-drug resistant plasma membrane glycoprotein levels were significantly increased. By contrast, silencing of Smad4, using stable RNA interference, inhibited TGFβ signaling, reversed the process of EMT and markedly increased the sensitivity of HCT116 cells to Dox. The results of the present study suggested that the combination of Dox with the downregulation of TGFβ signaling may be a potential novel therapeutic strategy with which to overcome chemoresistance during colon cancer chemotherapy.

## Introduction

Colon cancer is the third most common type of cancer worldwide and its incidence is increasing in East Asia and Western countries (1,2). Systematic chemotherapy is an important component of colon cancer treatment, particularly for patients with advanced disease (3). However, advances in chemotherapy for colon cancer have been limited as the underlying mechanisms causing chemoresistance remain to be elucidated. Doxorubicin (Dox) is extensively used as a chemotherapeutic agent in the treatment of colon cancer (4). Dox exerts its effect through interference with nucleoside metabolism, and nucleosides can be incorporated into RNA and DNA, leading to cytotoxicity and cell death (5). However, a significant difficulty associated with Dox treatment is the development of Dox-induced chemoresistance. An improved understanding of the ways in which resistance arises, and the molecular alterations that are associated with these processes, may result in the development of novel therapeutic strategies for the successful treatment of patients diagnosed with colon cancer.

Recent studies have suggested that epithelial-mesenchymal transition (EMT), metastasis and chemoresistance are closely associated with tumor progression (6,7). During the development of drug resistance, reversible epigenetic changes indicate alterations in the differentiation state of the tumor, which are likely to reflect EMT (8). The transforming growth factor β (TGFβ) family of cytokines are mediators of embryonic development and regulators of multiple types of physiological and pathophysiological EMT (9). RNA interference (RNAi) allows sequence-specific gene silencing. It involves small non-coding RNAs, which are associated with nuclease-containing regulatory complexes that then bind to complementary messenger RNA targets, thereby preventing the expression of these mRNAs (10).

The present study assessed whether the inhibition of TGFβ signaling represents a novel pathway regulating chemoresistance and the mechanisms underlying this effect.

## Materials and methods

### Cell culture

The HCT116 human colon cancer cell line was obtained from the Cell Bank of the Shanghai Institute of Biochemistry and Cell Biology, Chinese Academy of Sciences (Shanghai, China) and cultured in RPMI 1640 medium (Gibco-BRL, Gaithersburg, MD, USA) supplemented with 10% heat-inactivated fetal bovine serum, 10 U/ml penicillin, and 10 *μ*g/ml streptomycin in a humidified atmosphere containing 5% CO_2_ at 37°C.

### Reagents and antibodies

Dox was purchased from Aladdin Reagent Co., Ltd. (Shanghai, China). The primary polyclonal rabbit antibodies against human Smad4, Smad2, Smad3, phospho-Smad2 Ser465 (p-Smad2), phospho-Smad3 Ser425 (p-Smad3) and MDR p-gp (p-gp) were purchased from Cell Signaling Technology, Inc. (Beverly, MA, USA) and primary polyclonal rabbit antibodies against human E-cadherin (sc-7870), Vimentin (sc-5565), N-cadherin (sc-7939), Snai1 (sc-28199) and Slug (sc-15391) were purchased from Santa Cruz Biotechnology, Inc. (Dallas, TX, USA). These antibodies were used in western blot analysis and immunofluorescence staining at a dilution of 1:1,000. The TGFβ1 enzyme-linked immunosorbent assay (ELISA) kit was obtained from Invitrogen Life Technologies (Carlsbad, CA, USA). 3-(4,5-dimethylthazol-2-yl)-2,5-diphenyltetrazolium bromide (MTT) and other materials, including secondary antibodies were purchased from Beyotime Institute of Biotechnology (Shanghai, China).

### Lentiviral vector construction and transfection

The short hairpin RNA (shRNA)-encoding complementary single stranded oligonucleotides corresponding to Smad4 were designed as previously described (11). The RNAi sequence (5′-GTACTTCATACCATGCCGA-3′) was complementary to the human Smad4 cDNA sequence and was annealed and cloned into the pGCSIL-green fluorescent protein vector (GeneChem, Shanghai, China). The control sequence of RNAi (5′-TTCTCCGAACGTGTCACGT-3′) was used as a negative control. The recombinant virus was packaged using the Lentivector expression system (GeneChem). HCT116 cells were transfected with the recombinant lentivirus using Lipofectamine 2000 (Invitrogen Life Technologies) and transfected cells were screened under 800 *μ*g/ml G418 (Calbiochem, Darmstadt, Germany) for 4 weeks to generate stable monoclonal cell lines (Smad4 stable downregulation cell lines, RNAi-Smad4 and control stable cell lines, RNAi-NC). The expression of Smad4 was confirmed using reverse transcription-quantitative polymerase chain reaction (RT-qPCR) and western blot analysis.

### Drug treatment

Cells were divided into four groups, termed HCT116 cells, RNAi-NC cells, Smad4-RNAi cells and HCT116 cells untreated by Dox as a control (the control group). Cells were treated with 50 nmol/l Dox, or an equal volume of phosphate-buffered saline (PBS) for the controls, for 7 days (the medium was changed every alternate day, with the addition of the same dosage of Dox on occasion) for RT-qPCR, western blot analysis, immunofluorescence staining and ELISA.

### Western blotting

Following drug treatment, cells were washed twice with PBS and lysed in ice-cold radioimmunoprecipitation assay buffer (20 mM Tris-HCl, pH 7.4; 150 mM NaCl; 0.5% Nonidet P-40; 1 mM EDTA; 50 *μ*g/ml leupeptin; 30 *μ*g/ml aprotinin; 1 mM Na_3_VO_4_; and 1 mM phenylmeth-ylsulfonyl fluoride). The cell lysate (20 *μ*g) was separated on a sodium dodecyl sulfate polyacrylamide gel (SDS-PAGE) and then transferred onto a nitrocellulose membrane (Pall Corporation, Port Washington, NY, USA) by use of the wet transfer system (Bio-Rad, Hercules, CA, USA). Nonspecific binding sites were then blocked for 2 h at room temperature in Tris-buffered saline (TBS, pH 7.4) containing 0.1% Tween-20 and 10% bovine serum albumin (BSA). The primary antibodies were diluted (1:1,000) in TBS containing 0.1% Tween-20 and 5% BSA and incubated overnight at 4°C. The appropriate peroxidase-conjugated secondary antibodies (immunopure rabbit anti-goat IgG; goat anti-mouse IgG; and goat anti-rabbit IgG; Pierce Biotechnology, Inc., Rockford, IL, USA) were used at a dilution of 1:1,500. Positive antibody reactions were detected using SuperSignal West Pico Chemiluminescent Substrate (Pierce Biotechnology, Inc.).

### RT-qPCR

Total RNA was extracted using TRIzol reagent (Invitrogen Life Technologies) according to the manufacturer’s instructions and cDNA was synthesized from the mRNA obtained by RT-PCR using the SuperScript first-strand synthesis system (Fermentas, Vilnius, Lithuania). PCR was performed according to the standard protocol using a Roche LightCycler (Roche Diagnostics GmbH, Mannheim, Germany) with SYBR Green supermix (Bio-Rad). The PCR conditions were as follows: 31 Cycles of denaturation for 5 sec at 95°C, annealing for 30 sec at 60°C and primer extension for 60 sec at 72°C. cDNA was used as the template for the qPCR reaction with the following specific primers: Smad4, F 5′-TGTGACAGTGTCTGTGTGA-3′ and R 5′-CCTACCTGAACGTCCATTTC-3′; actin, F 5′-GTCCACCGCAAATGCTTCTA-3′ and R 5′-TGCTGTCACCTTCACCGTTC-3′. The human actin gene was amplified as an endogenous control. The gene expression of mRNA from each sample was calculated by normalizing against that of the reference gene, β-actin. Primer sequences are available upon request.

### MTT assay

The cell lines (HCT116, RNAi-NC and RNAi-Smad4) were seeded onto 96-well plates (6.0×10^3^ cells/well) and allowed to attach overnight. Following cellular adhesion, freshly prepared Dox at the appropriate concentration (10, 30, 50, 80 or 100 nmol/l), was added for the 24 h treatment, and 50 *μ*mol/l Dox was added for the 7 day treatment, respectively. The viability of the cells was evaluated using an MTT assay, according to the manufacturer’s instructions (Roche Applied Science, Indianapolis, IN, USA). Briefly, MTT was added at a concentration of 500 mg/l and the cells were incubated for 4 h at 37°C. The absorbance reading of each well was determined using a computer-controlled microtiter plate reader (iMARK; Bio-Rad) at a wavelength of 570 nm. The cell viability rates were defined as the relative absorbance of treated vs. untreated cells.

### Immunofluorescent staining

Cells were plated on 20 mm circular microscope coverslips. According to the manufacturer’s (Beyotime Institute of Biotechnology) instructions, cells were fixed in 4% paraformaldehyde for 15 min and permeabilized with 0.1% Triton X-100 for 15 min. Non-specific binding sites were then blocked for 1 h at room temperature in PBS (pH 7.4) containing 0.1% Tween-20 and 5% BSA. Primary antibodies (Snail and Slug, 1:200) in PBS containing 0.1% Tween-20 and 1% BSA were added overnight at 4°C in a humidified chamber. Cells were then incubated with the appropriate fluorescein isothiocyanate-conjugated secondary antibodies (1:200) for 1 h at room temperature in a humidified chamber in darkness. The samples were subsequently treated with 4′,6-diamidino-2-phenylindole (10 *μ*g/ml) for 30 sec to detect the cell nuclei. Images were obtained using fluorescence microscopy (BX53; Olympus, Tokyo, Japan) at magnification, ×400.

### ELISA

HCT116 cells, RNAi-NC cells and Smad4-RNAi cells were treated with 50 nmol/l Dox for 7 days and HCT116 cells, which were not subject to treatment with Dox were used as a control group. The concentration of TGFβ1 in the supernatant of the cells was measured using ELISA kits (Anogen-Yes Biotech Laboratories Ltd., Mississauga, ON, Canada) according to the manufacturer’s instructions.

### Statistical analysis

Statistical comparisons between the two groups were performed using an unpaired t-test. All groups were compared using a one-way analysis of variance, followed by Tukey’s post hoc test where appropriate. SPSS software, version 17.0 (SPSS, Inc., Chicago, IL, USA) was used for all analyses. P<0.05 was considered to indicate a statistically significant difference. All data are expressed as the mean ± standard deviation from at least three independent experiments.

## Results

### Dox treatment results in upregulation of TGFβ1 and phosphorylation of Smad2/3

**Figure 1:**
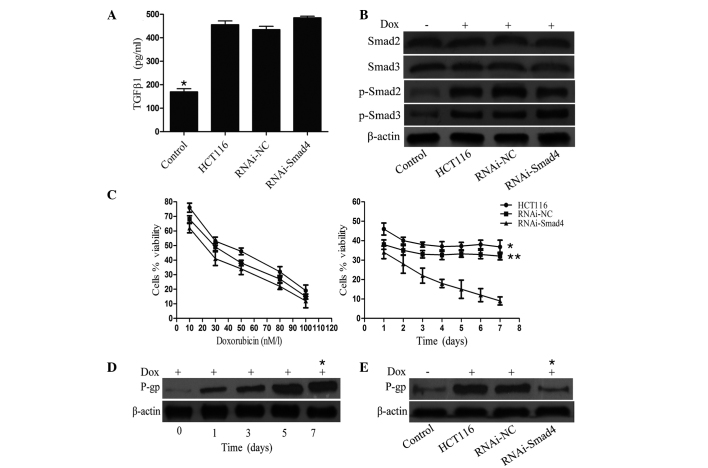
Changes in expression of molecules involved in the TGFβ/Smad4 signaling pathway, the cell viability ratio and p-gp expression, following Dox or Smad4 RNAi treatment. Expression of (A) TGFβ1, (B) Smad2/3 and p-Smad2/3 were examined using ELISA and western blotting. ^*^P<0.05 vs. HCT116 cells (P=0.022), RNAi-NC cells (P=0.018) and Smad4-RNAi cells (P=0.013). (C) Cell viability ratio was assessed using an MTT assay. Data are presented as a percentage of the control cell viability. (D) and (E) p-gp level was investigated using a western blot assay, with significant differences observed between p-gp expression levels at 0 and 7 days (^*^P=0.029), and between RNAi-Smad4 vs. HCT116 cells (^*^P=0.004) and RNAi-NC cells (^*^P=0.007). β-actin expression was used as a reference gene. TGF, transforming growth factor; Dox, doxorubicin; p-gp, multi-drug resistant plasma membrane glycoprotein; RNAi, RNA interference; NC, normal control.

Dox has been observed to induce expression of circulating TGFβ in xenograft and transgenic animal models (12,13). In order to identify whether and how the TGFβ/Smad4 signaling pathway was affected by Dox administration, the effects of Dox treatment on TGFβ1 concentration and p-Smad2/Smad3 expression level in HCT116 cells were examined using ELISA and western blotting. Following treatment with 50 nmol/l Dox for 7 days, it was shown that the expression of TGFβ1 in HCT116 cells was significantly higher (P<0.05) than that in the control (HCT116 cells treated with PBS; Fig. 1A). Similarly, the levels of p-Smad2 and Smad3 in the HCT116 cells increased in comparison with the control group (Fig. 1B). Thus, the present results indicated that long-term administration of Dox may activate the TGFβ/Smad4 pathway in HCT116 cells.

### HCT116 cells exhibit resistance following long-term administration of Dox

To further examine the effects of Dox on HCT116 cells, the cell viability and p-gp levels were assessed using an MTT assay and western blotting. HCT116 cells were treated with 10, 30, 50, 80 and 100 nmol/l Dox for 24 h, and with 50 nmol/l Dox for 1, 2, 3, 4, 5, 6 and 7 days, respectively. Cells treated with PBS alone were used as a control. The results from the MTT assay indicated that the cell viability of HCT116 cells decreased in a dose-dependent manner (Fig. 1C). A concentration of 50 nmol/l was determined to be appropriate for long-term Dox administration for the MTT assay. It was observed that the viability of HCT116 cells decreased between 1 and 3 days in a time-dependent manner. However, it did not change markedly between 3 and 7 days, following treatment (Fig. 1C). Following administration of 50 nmol/l Dox, the p-gp expression of HCT116 cells increased in a time-dependent manner (Fig. 1D). These data demonstrated that HCT116 cells acquired resistance to Dox following long-term treatment at a low concentration.

### Expression of Smad4 is significantly downregulated using a lentivirus vector

**Figure 2:**
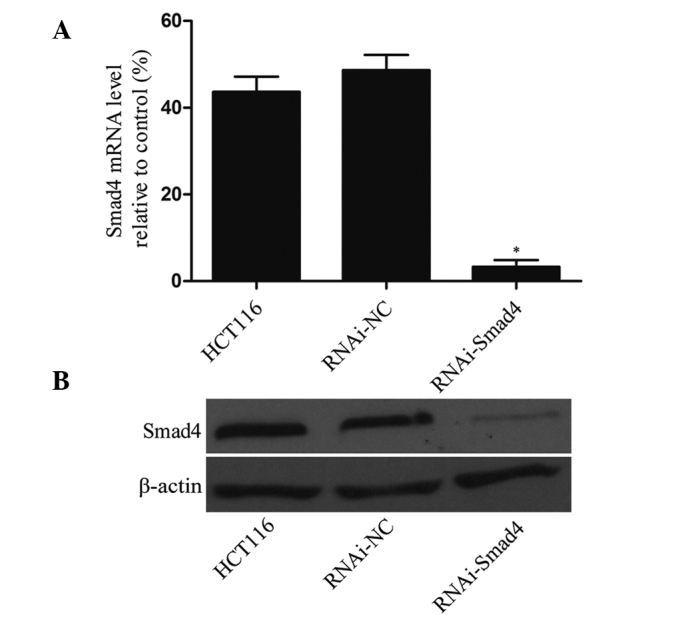
Effect of lentiviral-mediated short hairpin RNA impact on Smad4 expression levels. Expression of Smad4 (A) mRNA and (B) protein was detected using reverse transcription-quantitative polymerase chain reaction and western blotting. β-actin expression was used as a reference gene for mRNA and protein. ^*^P<0.01 vs. HCT116 cells and RNAi-NC cells. RNAi, RNA interference; NC, normal control.

In order to assess the knockdown efficiency of the lentiviral vector, the mRNA and protein expression of Smad4 in HCT116 cells, RNAi-NC cells and Smad4-RNAi cells was assessed. The results of the RT-qPCR and western blotting experiments revealed that mRNA and protein levels of Smad4 in Smad4-RNAi cells were significantly lower than that in HCT116 cells or RNAi-NC cells (P<0.01), while no significant difference was observed between HCT116 cells and RNAi-NC cells (P>0.05; Fig. 2).

### HCT116 cells exhibit molecular changes consistent with EMT following Dox treatment

**Figure 3:**
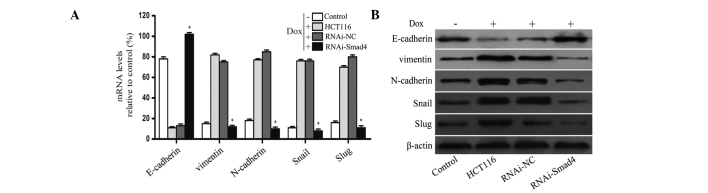
HCT116 cells exhibit changes in EMT markers and transcription factors, following Dox or Smad4-specific RNAi treatment. Expression of EMT markers and transcription factors was analyzed using (A) reverse transcription-quantitative polymerase chain reaction and (B) western blotting. β-actin expression was used as a reference gene for mRNA and protein expression. ^*^P<0.05 vs. HCT116 cells and RNAi-NC cells. EMT, epithelial-mesenchymal transition; RNAi, RNA interference; NC, normal control.

**Figure 4:**
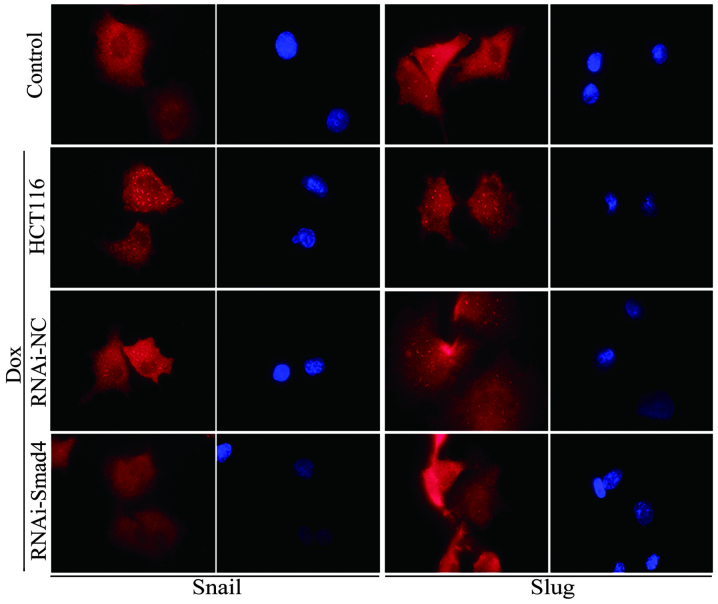
Immunofluorescence staining to detect the expression and cellular localization of Snail and Slug. Cell nuclei staining (blue) was performed as a control. Magnification, ×400. RNAi, RNA interference; NC, normal control; Dox, doxorubicin.

To determine whether the acquisition of Dox resistance was associated with specific molecular changes that are consistent with EMT, the expression of epithelial and mesenchymal phenotypic markers, following 50 nmol/l Dox administration, were examined using RT-qPCR and western blotting. From the PCR data, a significant reduction in the expression of E-cadherin, and an upregulation of Vimentin and N-cadherin, in HCT116 cells was observed compared with that in the control group. The expression of the transcription factors, Snail and Slug, were significantly increased in resistant cells and the western blotting results were in accordance with the PCR data (Fig. 3A and B). Furthermore, immunofluorescence staining revealed that Snail and Slug were more localized in the cytoplasm of the HCT116 cells than it was in the control group (Fig. 4). These results suggested that EMT occurred in HCT116 cells following treatment with Dox.

### Downregulation of Smad4 reverses Dox-induced EMT

Following successful silencing of the Smad4 gene using a lentiviral vector, Smad4-specific RNAi was used to investigate the effect of inhibiting the TGFβ/Smad4 signaling pathway on the process of EMT, which was induced following Dox treatment. RNAi-NC cells and Smad4-RNAi cells were also treated with 50 nmol/l Dox for 7 days, and the expression of EMT markers and transcription factors were examined using RT-qPCR and western blotting. The PCR data demonstrated that the expression of E-cadherin in Smad4-RNAi cells was higher than that observed in the RNAi-NC cells (P<0.05), and that the expression levels of Vimentin, N-cadherin, Snail and Slug in Smad4-RNAi cells was significantly lower than that detected in the RNAi-NC cells (P<0.05). The western blotting results were in accordance with the PCR data (Fig. 3A and B). Furthermore, immunofluorescence staining revealed that in the RNAi-NC cells, Snail and Slug were more localized to the cytoplasm than they were in the Smad4-RNAi cells (Fig. 4). These results suggested that downregulation of Smad4 reverses EMT, which is induced following Dox treatment.

### Downregulation of Smad4 enhances and increases sensitivity of HCT116 cells to Dox

In order to examine the effect of the downregulation of Smad4 on HCT116 cell sensitivity to Dox, the cell viability and MDR p-gp levels were also investigated using an MTT assay and western blot analysis, in RNAi-NC cells and Smad4-RNAi cells. Following administration of the same treatment to HCT116 cells, the MTT assay results indicated that the cell viability ratio of RNAi-NC and Smad4-RNAi cells decreased in a dose-dependent manner (Fig. 1C). Concerning the MTT data from the long-term administration of 50 nmol/l Dox, it was shown that the cell viability ratio of RNAi-NC cells decreased between 1 and 3 days following treatment, in a time-dependent manner, whilst it did not alter markedly between 3 and 7 days following treatment. This result was similar to that obtained in the HCT116 cells. However, the cell viability ratio of Smad4-RNAi cells decreased in a time-dependent manner for the duration of the experiment (Fig. 1C). Following 7 days of 50 nmol/l Dox administration, the p-gp expression of RNAi-NC cells was higher than that in the control and Smad4-RNAi cells (Fig. 1E). These data suggested that downregulation of Smad4 increases HCT116 cell sensitivity to Dox following long-term treatment at a low concentration.

## Discussion

There are two primary forms of drug resistance that are associated with chemotherapy used in cancer. Patients who are initially refractory to therapy exhibit intrinsic drug resistance, while patients who relapse following an initial response to the therapy, do so as a result of acquired drug resistance (14). Previous studies have demonstrated that resistance to Dox treatment may be due to the activation of expression of the MDR p-gp gene (15) and activation of intracellular signaling pathways (16,17). Despite advances in treatment, chemoresistance remains a significant impediment to the treatment of colon cancer. An improved understanding of the mechanisms by which residual tumor cells survive following chemotherapy may ultimately provide novel, effective chemotherapeutic strategies. In the present study, HCT116 human colon cancer cells were used to investigate the molecular mechanisms underlying Dox resistance and the associated cellular behaviors.

It was shown that HCT116 cells underwent changes associated with EMT, following long-term Dox treatment. This was demonstrated by changes in the expression of molecular markers and proteins, including decreased expression of E-cadherin, and increased levels of Vimentin and N-cadherin, as elucidated using RT-qPCR and western blot analysis. These changes correlated with a significant increase in the expression of transcription factors Snail and Slug as elucidated using RT-qPCR, western blot analysis and immunofluorescence staining. In accordance with the present findings, the induction of EMT has also been reported in acquired resistance to other chemotherapeutic agents (18,19). From the MTT and western blotting data in the present study, it was observed that HCT116 cells exhibited induced resistance to Dox, as shown by the reduction in the cell viability ratio and the increase in p-gp expression, in association with EMT. It was hypothesized that this may be associated with the upregulation of TGFβ1 expression, and the phosphorylation of Smad2 and Smad3, which was triggered by treatment with Dox.

TGFβ is a ubiquitous, pleiotropic growth factor, which regulates numerous cellular processes, including cell proliferation, differentiation and apoptosis (20). There are three TGFβ isoforms, TGFβ1, TGFβ2 and TGFβ3, which exert their effects by binding to cell surface receptors; the type I (TβRI), type II (TβRII) and type III (TβRIII) TGFβ receptors. TβRII is a constitutively active serine/threonine kinase that, upon ligand binding, recruits and phosphorylates TβRI, thereby stimulating TβRII serine/threonine kinase activity. TβRI then phosphorylates and activates the transcription factors Smad2 or Smad3, which form a complex with Smad4. This complex translocates to the nucleus and initiates the transcription of TGFβ target genes in a cell-specific manner (21). Thus, silencing the Smad4 gene may block TGFβ signaling and inhibit its molecular and biological effects. Lim *et al* (22) utilized knockdown of Smad4 in order to block TGFβ signaling in lung cells. A previous study confirmed that the TGFβ/Smad4 pathway has an important role in the chemoresistance of colon cancer cells to Dox-induced cell death (23). EMT is a latent process, important during embryonic development, by which certain cells within a tumor may reactivate mesenchymal traits to disperse and form metastases in the process of cancer progression (24). Induction of EMT by TGFβ has been observed to increase motility and chemoresistance via the disassembly of cell-to-cell contacts, loss of cell polarity and significant cytoskeletal reorganization in normal and malignant mammary epithelial cell types (25,26).

In accordance with previous studies (27,28), the present data indicated that downregulation of Smad4 may be a crucial event in the reversal of Dox-induced EMT. The current results demonstrated that Smad4 RNAi reversed the changes in the expression of the EMT markers E-cadherin, Vimentin and N-cadherin, and that of the EMT transcription factors, Snail and Slug, which were induced by Dox, indicating EMT reversal. Concomitantly, Smad4 RNAi resulted in a persistent reduction in the viability and MDR p-gp expression of cells treated with Dox. Although further studies are required to elucidate the mechanisms underlying chemoresistance, the use of Smad4 shRNA during chemotherapy may be a potential therapeutic approach with which to improve treatment efficacy. As hypothesized, knockdown of Smad4 did not significantly affect the expression of TGFβ1, Smad2 or Smad3, or the phosphorylation of Smad2/3, as they are located upstream of the TGFβ/Smad4 pathway, in contrast to Smad4.

In conclusion, the present study demonstrated that low concentration, long-term administration of Dox may promote resistance in HCT116 colon cancer cells, in part via the activation of TGFβ signaling. In turn, this triggered Vimentin, N-cadherin, Snail and Slug expression, indicative of the occurrence of EMT. The present study also suggested that knockdown of Smad4 to inhibit the TGFβ signal during chemotherapy may sensitize cancer cells to chemotherapy, in part through the inhibition of MDR p-gp expression and reversal of the EMT process. This may result in increased therapeutic efficacy and the need for lower doses of chemotherapeutic agents. Therefore, downregulation of Smad4, or treatment with inhibitors to inhibit TGFβ signaling or Smad2 and/or Smad3 phosphorylation, combined with Dox may be a potential novel strategy with which to treat colon cancer.

## References

[1] Ferlay J, Steliarova-Foucher E, Lortet-Tieulent J (2013). Cancer incidence and mortality patterns in Europe: Estimates for 40 countries in 2012. Eur J Cancer.

[2] Cui R, Okada Y, Jang SG (2011). Common variant in 6q26-q27 is associated with distal colon cancer in an Asian population. Gut.

[3] Jonker DJ, Spithoff K, Maroun J (2011). Adjuvant systemic chemotherapy for stage II and III colon cancer after complete resection: An updated practice guideline. Clin Oncol (R Coll Radiol).

[4] Colombo V, Lupi M, Falcetta F, Forestieri D, D Incalci M, Ubezio P (2011). Chemotherapeutic activity of silymarin combined with doxorubicin or paclitaxel in sensitive and multidrug-resistant colon cancer cells. Cancer Chemoth Pharm.

[5] Arafa el-Sa, Zhu Q, Shah ZI (2011). Thymoquinone up-regulates PTEN expression and induces apoptosis in doxorubicin-resistant human breast cancer cells. Mutat Res.

[6] Rosanò L, Cianfrocca R, Spinella F (2011). Acquisition of chemoresistance and EMT phenotype is linked with activation of the endothelin a receptor pathway in ovarian carcinoma cells. Clin Cancer Res.

[7] Bastid J (2012). EMT in carcinoma progression and dissemination: Facts, unanswered questions, and clinical considerations. Cancer Metast Rev.

[8] Canino C, Mori F, Cambria A (2012). SASP mediates chemoresistance and tumor-initiating-activity of mesothelioma cells. Oncogene.

[9] Borthwick LA, Gardner A, De Soyza A, Mann DA, Fisher AJ (2012). Transforming growth factor-β1 (TGF-β1) driven epithelial to mesenchymal transition (EMT) is accentuated by tumour necrosis factor α (TNFα) via crosstalk between the SMAD and NF-κB pathways. Cancer Microenviron.

[10] Davidson BL, McCray PB (2011). Current prospects for RNA interference-based therapies. Nat Rev Genet.

[11] Huang X, Huang S, Zhang F (2010). Lentiviral-mediated Smad4 RNAi promotes SMMC-7721 cell migration by regulation of MMP-2, VEGF and MAPK signaling. Mol Med Rep.

[12] Lindner D (2014). Animal models and the tumor microenvironment: Studies of tumor-host symbiosis. Semin Oncol.

[13] Nakasone ES, Askautrud HA, Kees T (2012). Imaging tumor-stroma interactions during chemotherapy reveals contributions of the microenvironment to resistance. Cancer Cell.

[14] Gottesman MM (2002). Mechanisms of cancer drug resistance. Annu Rev Med.

[15] Xiong XB, Lavasanifar A (2011). Traceable multifunctional micellar nanocarriers for cancer-targeted co-delivery of MDR-1 siRNA and doxorubicin. ACS Nano.

[16] Ghosh J, Das J, Manna P, Sil PC (2011). The protective role of arjunolic acid against doxorubicin induced intracellular ROS dependent JNK-p38 and p53-mediated cardiac apoptosis. Biomaterials.

[17] Sims JT, Ganguly SS, Bennett H, Friend JW, Tepe J, Plattner R (2013). Imatinib reverses doxorubicin resistance by affecting activation of STAT3-Dependent NF-κB and HSP27/p38/AKT pathways and by inhibiting ABCB1. PloS one.

[18] Li QQ, Chen ZQ, Cao XX (2011). Involvement of NF-κB/miR-448 regulatory feedback loop in chemotherapy-induced epithelial-mesenchymal transition of breast cancer cells. Cell Death Differ.

[19] Sun L, Yao Y, Liu B (2012). MiR-200b and miR-15b regulate chemotherapy-induced epithelial-mesenchymal transition in human tongue cancer cells by targeting BMI1. Oncogene.

[20] Tian M, Neil JR, Schiemann WP (2011). Transforming growth factor-β and the hallmarks of cancer. Cell Signal.

[21] Javelaud D, Alexaki VI, Dennler S, Mohammad KS, Guise TA, Mauviel A (2011). TGF-β/SMAD/GLI2 signaling axis in cancer progression and metastasis. Cancer Res.

[22] Lim MJ, Lin T, Jakowlew SB, Cheng Y (2012). Signaling mechanisms of transforming growth factor-β (TGF-β) in cancer: TGF-β induces apoptosis in lung cells by a Smad-dependent mechanism. Tumor Suppressor Genes.

[23] Kang Y, Park M, Heo S (2013). The radio-sensitizing effect of xanthohumol is mediated by STAT3 and EGFR suppression in doxorubicin-resistant MCF-7 human breast cancer cells. Biochim Biophys Acta.

[24] Micalizzi DS, Farabaugh SM, Ford HL (2010). Epithelial-mesenchymal transition in cancer: Parallels between normal development and tumor progression. J Mammary Gland Biol.

[25] Smith AL, Iwanaga R, Drasin DJ (2012). The miR-106b-25 cluster targets Smad7, activates TGF-β signaling, and induces EMT and tumor initiating cell characteristics downstream of Six1 in human breast cancer. Oncogene.

[26] Fuxe J, Vincent T, de Herreros AG (2010). Transcriptional crosstalk between TGFβ and stem cell pathways in tumor cell invasion: Role of EMT promoting Smad complexes. Cell Cycle.

[27] Hesling C, Fattet L, Teyre G (2011). Antagonistic regulation of EMT by TIF1γ and Smad4 in mammary epithelial cells. Embo Rep.

[28] Wang Y, Li W, Zang X (2013). MicroRNA-204-5p regulates epithelial-to-mesenchymal transition during human posterior capsule opacification by targeting SMAD4. Invest Ophth Vis Sci.

